# Quality of cervical cancer screening in Brazil: external assessment of the PMAQ

**DOI:** 10.1590/S1518-8787.2017051006802

**Published:** 2017-07-07

**Authors:** Mara Rejane Barroso Barcelos, Rita de Cássia Duarte Lima, Elaine Tomasi, Bruno Pereira Nunes, Suele Manjourany Silva Duro, Luiz Augusto Facchini

**Affiliations:** I Departamento de Ginecologia e Obstetrícia. Centro de Ciências da Saúde. Universidade Federal do Espírito Santo. Vitória, ES, Brasil; II Departamento de Enfermagem. Centro de Ciências da Saúde. Universidade Federal do Espírito Santo. Vitória, ES, Brasil; III Departamento de Medicina Social. Faculdade de Medicina. Universidade Federal de Pelotas. Pelotas, RS, Brasil; IV Departamento de Enfermagem. Faculdade de Enfermagem. Universidade Federal de Pelotas. Pelotas, RS, Brasil

**Keywords:** Uterine Cervical Neoplasms, diagnosis, Socioeconomic Factors, Women’s Health Services, Program Evaluation

## Abstract

**OBJECTIVE:**

To examine whether demographic and socioeconomic variables and the variables of the organization of services are associated with the quality of cervical cancer screening.

**METHODS:**

This is a survey carried out in the health services of the five Brazilian regions in 2012. The sample consisted of users of basic health units participating in the Program for Improving Access and Quality of the Primary Care. The independent variables analyzed were: socioeconomic characteristics (municipal context), demographic characteristics (user profile), and two domains related to the organization of basic services (work structure and process). The low quality of the screening was assessed from the lack of access, late examination, and lack of guidance. Crude and adjusted analyses by Poisson regression assessed the association between outcomes and independent variables.

**RESULTS:**

The values of lack of access, late examination, and lack of guidance were 6.7%, 11.2%, and 19.2%, respectively. Problems of quality were lower according to the increase in Municipal Human Development Index and per capita household income, increasing with population size and municipal coverage of the Family Health Strategy. The Midwest region of the country presented the highest occurrences of low quality outcomes. Indigenous and yellow women had the highest prevalence of outcomes. Women with partner, who received the *Bolsa Família* Program, and who had paid work had less chances of having lack of access, late examination, and lack of guidance. The appropriate work process in health services decreased the likelihood of low quality in all indicators.

**CONCLUSIONS:**

Investments in the work process of health teams, social cash transfer programs, and social conditions of the population are essential to improve the quality of the program of cervical cancer screening in Brazil.

## RESUMO

**OBJETIVO:**

Analisar se as variáveis demográficas, socioeconômicas e da organização dos serviços estão associadas à qualidade do rastreamento do câncer de colo uterino.

**MÉTODOS:**

Inquérito realizado em serviços de saúde das cinco regiões brasileiras em 2012. A amostra foi composta por usuárias de unidades básicas de saúde participantes do Programa de Melhoria do Acesso e da Qualidade na Atenção Básica. As variáveis independentes analisadas foram: características socioeconômicas (contexto municipal); características demográficas (perfil de usuárias); e dois domínios relativos à organização dos serviços básicos (estrutura e processo de trabalho). A baixa qualidade do rastreamento foi avaliada por meio da falta de acesso, atraso na realização do exame e falta de recebimento de orientações. Análises bruta e ajustada por meio de regressão de Poisson avaliaram a associação entre os desfechos e as variáveis independentes.

**RESULTADOS:**

A falta de acesso, atraso na realização do exame e falta de recebimento de orientações foram de 6,7%, 11,2% e 19,2%, respectivamente. Os problemas de qualidade foram menores de acordo com o aumento do Índice de Desenvolvimento Humano Municipal e da renda familiar *per capita*, aumentando com o porte populacional e a cobertura municipal da Estratégia Saúde da Família. A região Centro-Oeste do país apresentou as maiores ocorrências dos desfechos de baixa qualidade. As mulheres de raça indígena e amarela tiveram as maiores prevalências dos desfechos. As mulheres com companheiro, que recebiam o benefício do Programa Bolsa Família e tinham trabalho remunerado tiveram menos falta de acesso, menos atraso na realização do exame e menos falta de recebimento de orientações. O processo de trabalho adequado nos serviços de saúde diminuiu a probabilidade de baixa qualidade em todos os indicadores.

**CONCLUSÕES:**

Investimentos em processo de trabalho das equipes de saúde, programas sociais de transferência de renda e condições sociais da população são essenciais para melhorar a qualidade do programa de rastreamento de colo de útero no Brasil.

## INTRODUCTION

Cervical cancer is an important public health problem, being the third most frequent and the fourth cause of mortality in women in Brazil, with an annual incidence of 16,340 cases, estimated risk of 15.85 cases per 100,000 women, and mortality rate of 4.86 cases per 100,000 women^9^.

According to the parameters of the World Health Organization (WHO), the estimated incidence of this cancer should be up to 16.3 cases per 100,000 women and, for mortality, 7.3 per 100,000 women. The challenge of Brazil is to reduce them further, using as references Western Asia, with incidence of 4.4 cases per 100,000 women, as well as Australia and New Zealand, with incidences of 5.5 cases per 100,000 women, which, along with Western Europe, have death rates of less than two per 100,000 women^28^.

In this context, care actions become relevant for women’s health and cervical cancer screening, priorities for the Brazilian Unified Health System (SUS) and the Brazilian research agenda^a^. After the implementation of population-based screening programs in developed countries, there was a reduction in mortality from cervical cancer^26^ ^,^ ^28^.

For the screening of the problem, the WHO recommends that at least 80% of the women aged between 25 and 64 years, who have already begun sexual activity, perform the cervical Pap smear test every three years, after two consecutive annual negative controls^27^. In Brazil, the *Plano de Enfrentamento de Doenças Crônicas* 2011–2022 (2011–2022 Plan for Combating Chronic Diseases)^17^ set a goal of 85% for the coverage of the exam. Screening in this age group is justified by the increased occurrence of high grade lesions, which can have effective treatment, preventing its evolution into cancer^9^. Among the criteria for the screening test, we can mention security, easy acceptance, proven sensitivity and specificity, and good cost-effectiveness^9^, which are found in the Pap smear test.

Coinciding with the implementation and consolidation of the SUS and the Family Health Strategy (FHS), Brazilian estimates show that the coverage of the Pap smear increased from 82.6% in 2003 to 87.6% in 2008^7^, achieving international and national goals^15^ ^,^ ^26^ ^,^ ^27^. Despite the contributions of the primary health care in the expansion of the coverage and offer of cervical cancer screening and control in Brazil^4^, several studies have observed smaller coverage among women with higher social vulnerability, especially in the poorest regions of the country^11^ ^,^ ^16^. The social inequities observed suggest the need for a better understanding of the reasons underlying the high incidence and mortality rates from cervical cancer in relation to the high provision and coverage of a procedure for potentially effective early diagnosis. Problems in the quality of collection procedures, the agility of results, and the timely treatment of lesions^17^, often arising from shortcomings in the work structure and process of primary care services^24^, may negatively reflect on the occurrence of cervical cancer, requiring further deepening with comprehensive studies.

In this sense, we intended to address the quality of cervical cancer screening of the basic health network across Brazil, using data from the *Programa de Melhoria do Acesso e da Qualidade da Atenção Básica* (PMAQ – Program for Improving Access and Quality of the Primary Care). This program encourages the improvement of access and quality practices and consists of the stages of adherence, contracting, development, external assessment, and recontracting^b^. This study is part of the external assessment of the PMAQ, with particular emphasis on the services of the FHS^b^.

Seeking to better understand the problem and support the effectiveness of the primary health care, this study analyzes the relationship between the socioeconomic (municipal context) and demographic (user profile) variables, two domains related to the organization of basic services (work structure and process), and three outcomes selected to assess the quality of the cervical cancer screening in users of Basic Health Units (BHU) participating in the PMAQ.

## METHODS

This is a survey on health services, being the first cycle of the external assessment of the PMAQ. The scenario in this study was the BHU, with voluntary adherence of the teams to the program. We assessed, using a census of the structure of services, 38,812 BHU, located in all Brazilian municipalities. Additionally, we studied the work process of 17,202 primary health care teams, located in 3,965 municipalities (71.3% of the total) that joined the PMAQ, and we interviewed 65,391 individuals (approximately four users per team). For this study, we selected 35,844 users within the teams assessed, which represented the total of women participating in the study aged between 25 and 64 who used the health services, regardless of reason. In each team, a health professional with higher education provided information about the characteristics of the work structure and process of the service. The 17,202 teams assessed were located in 13,843 BHU, selected for this study. We did not include the remaining 24,969 BHU for which information was available only on the structure of services.

Data collection occurred between June 2012 and March 2013 from the application of electronic questionnaires, available in tablets, by approximately 1,000 interviewers and supervisors selected after qualification. The tablets were essential to give agility to the process and ensure the quality of the research data. The quality control strategy involved the supervision of the data collection process, the use of an electronic validator, the checking of the consistency of each question, the checking of the consistency between answers, and the control of the time of the interview by comparing their start and end times.

To meet the objectives of this article, we estimated sample size considering the following parameters: a significance level of 5%, 80% power, rate of those not exposed to those exposed of 1:6, and prevalence of outcome of 55% among those not exposed. To detect prevalence ratios starting from 1.1, we estimated a sample of 5,325 women. By adding 10% for losses and refusals and 15% for the control of confounding factors, the sample size required was 6,735 women.

The survey instrument was comprised of three modules: Module I – health unit observation; Module II – interview with the primary care professional team and verification of documents in the health unit; and, Module III – interview with users in the health unit^b^.

In Module I, the structure of the BHU was characterized by direct observation of the interviewers. In Module II, we assessed the work process of the team, interviewing a professional with higher education from each health team. In Module III, we interviewed the users who were at the BHU on the day of the external assessment and who had used the services of the unit at least once in the year prior to the interview.

The outcomes of this study measured problems in the quality of cervical cancer screening, from three indicators: proportion of women aged 25 to 64 years interviewed at the BHU who had never performed the examination (lack of access throughout life), cervical cancer screening that was performed more than 36 months ago (late examination), and no guidance on the importance of the preventive examination for cervical cancer and the period for new collection (lack of guidance).

The independent variables included the characteristics of the social context, the individuals, and the work structure and process of the BHU. For context, we selected the Municipal Human Development Index (MHDI) with distribution according to quartiles (up to 0.658, 0.659 to 0.750, 0.751 to 0.806, 0.807 to 0.819), population size of the municipalities (up to 30,000, 30,001 to 100,000, 100,001 to 500,000, over 500,000 inhabitants), FHS coverage (up 29.9%, 30% to 64.9%, 65% or over), and geographic region (North, Northeast, Midwest, Southeast, South). The MHDI is a marker of wealth of municipalities, consisting of data on life expectancy at birth, education, and per capita Gross Domestic Product (GDP), ranging from zero (no human development) to one (total human development), categorized into quartiles^7^.

Population size of the municipality can be considered a marker of the conditions of wealth and infrastructure available^5^. The FHS coverage is taken in this study as a proxy of the governance decision in favor of extending basic health services capable of promoting greater equity in care, favoring vulnerable territories and populations^5^.

Individual variables were: age in full years (25 to 35, 36 to 49, 50 to 64), self-reported race according to the categories of the Brazilian Institute of Geography and Statistics (IBGE) (white, black, brown, yellow, indigenous), marital status (no partner; with partner), number of residents (1 to 3, 4 to 5, 6 or more), being part of the *Bolsa Família* Program (no, yes), paid work (no, yes), and per capita family income in quartiles (up to R$104, R$105 to R$188, R$189 to R$300, R$301 or over). The number of residents in the household, being part of the *Bolsa Família* Program, and the per capita family income are markers of the social condition of users.

In relation to the structure for the development of screening actions, we considered as appropriate the availability of all ten inputs and materials: gynecological examination table, spotlight, gloves, disposable speculum, endocervical brush, Ayre spatula, slide clamp, glass slide, plastic jar with lid, and request record of the *Sistema de Informações do Câncer do Colo do Útero* (SISCOLO – Information System of Cervical Cancer)^c^. The presence of seven to nine inputs was classified as less adequate, and the availability of six or fewer inputs was classified as inadequate. As for the work process, we considered as adequate the reference of all six actions of the team: collection of cervical cytology, record of women with late Pap smear, record of altered Pap smear, follow-up of women after treatment, use of dissemination and awareness strategies for examination, and provision of educational and health promotion actions focused on cervical cancer. The mention of four to five actions was classified as less adequate, and three or fewer actions were classified as inadequate.

Stata 13,0 (College Station, TX) was used for all data analysis. We carried out the description of the frequency distribution and the bivariate analysis between the outcomes and the contextual and individual variables and the variables of the characteristics of health services using the Chi-square test for heterogeneity (nominal categorical and dichotomous variables) and linear trend (ordinal categorical variables). For the crude and adjusted analyses, we use Poisson regression with robust variance according to the independent variables, and the statistical significance was obtained using Wald test for heterogeneity and linear trend, according to the type of each variable. In the adjusted analysis, we adopted a hierarchical model consisting of three levels: distal, with the characteristics of the social context (MHDI, population size, FHS coverage, and geographic region); intermediate, grouping the demographic and socioeconomic characteristics of users (age, race, marital status, number of residents, *Bolsa Família* benefit, paid work, and per capita income); and, proximal, with the aspects of health services (structure and team work process) directly involved with the performance of cervical cancer screening. In each of the hierarchical levels, we carried out the backward regression and we kept all the variables with p-value ≤ 0.20 in the model. For all analyses, we considered a significance level of 5%.

The project of external assessment of the PMAQ was submitted to the Research Ethics Committee of the Universidade Federal de Pelotas (Number 38/2012, May 10, 2012). An informed consent was obtained from all users interviewed.

## RESULTS

The total of 35,844 users aged 25 to 64 years was located at 17,202 health teams that were installed in 13,843 BHU. Of the total of BHU studied, 62.3% had only one health team. According to Table 1, compliance to the study showed difference between regions, being higher in the Southeast (37.1%) and lower in the North (5.6%). Loss of information was small for the variables under study, except for per capita income. The missing data for the variables of MHDI, population size, FHS coverage, and geographic region was 2% of the total. The variables of age, marital status, *Bolsa Família*, and paid work showed no missing data (0%). The variable of race had missing data of 0.8%; the number of residents, 0.3%; and, per capita income, 16.7%. The variable of adequacy of the structure had missing data of 2.3% and adequacy of the process, 3.4%.

**Table 1:** Description of the sample and bivariate analyses between outcomes and contextual and individual variables and the variables of characteristics of health services. PMAQ, Brazil, 2012.

Variable	Distribution of the sample		Lack of access throughout life		Examination carried out more than 36 months ago		No guidance on the examination	
			
	n	%	% (95%CI)	p	% (95%CI)	p	% (95%CI)	p
MHDI				< 0.001*		< 0.001*		0.001*
Up to 0.658	8,867	25.2	8.7 (8.1–9.3)		13.2 (12.5–13.9)		19.2 (18.4–20.1)	
0.659 to 0.75	8,813	25.1	7.0 (6.4–7.6)		11.3 (10.6–12.0)		19.8 (18.9–20.6)	
0.751 to 0.806	8,713	24.8	5.8 (5.3–6.3)		10.6 (9.9–11.2)		19.3 (18.5–20.1)	
0.807 to 0.919	8,738	24.9	4.7 (4.3–5.2)		9.3 (8.6–9.9)		17.8 (17.0–18.6)	
Population size (inhabitants)				< 0.001*		0.001*		< 0.001*
Up to 30,000	14,744	42.0	7.3 (6.8–7.7)		11.6 (11.0–12.1)		17.9 (17.3–18.6)	
30,001 up to 100,000	7,445	21.2	6.8 (6.2–7.4)		11.5 (10.8–12.3)		19.1 (18.2–20.0)	
100,001 up to 500,000	6,422	18.3	5.6 (5.1–6.2)		10.4 (9.6–11.1)		20.1 (19.2–21.3)	
500,001 or more	6,520	18.6	5.7 (5.1–6.3)		10.2 (9.5–11.0)		20.3 (19.3–21.3)	
FHS coverage (%)				< 0.001*		< 0.001*		0.080
Up to 29.9	2,858	8.1	5.5 (4.7–6.3)		9.4 (8.3–10.5)		18.8 (17.3–20.2)	
30 to 64.9	11,696	33.3	5.6 (5.2–6.1)		10.2 (9.6–10.7)		18.5 (17.8–19.2)	
65 or more	20,577	58.6	7.3 (6.9–7.6)		11.9 (11.4–12.3)		19.4 (18.8–19.9)	
Geographic region				< 0.001		< 0.001		< 0.001
North	1,955	5.6	7.8 (6.6–9.0)		11.5 (10.1–13.0)		22.4 (20.5–24.3)	
Northeast	12,897	36.7	7.7 (7.2–8.1)		12.0 (11.5–12.6)		19.6 (18.9–20.3)	
Southeast	13,026	37.1	5.8 (5.4–6.2)		10.4 (9.9–11.0)		18.3 (17.6–19.0)	
South	5,178	14.7	4.6 (4.1–5.2)		9.2 (8.4–10.0)		16.1 (15.1–17.2)	
Midwest	2,075	5.9	8.6 (7.4–9.8)		13.7 (12.1–15.2)		23.7 (21.9–25.6)	
Age (years)				< 0.001*		0.001*		< 0.001*
25–35	13,397	37.4	8.3 (7.8–8.8)		12.1 (11.6–12.7)		20.6 (19.9–21.3)	
35–49	11,846	33.1	4.8 (4.4–5.2)		9.1 (8.6–9.6)		16.6 (15.9–17.2)	
50–64	10,601	29.6	6.6 (6.1–7.1)		12.3 (11.7–12.9)		20.2 (19.4–21.0)	
Race				< 0.001		< 0.001		< 0.001
White	13,197	37.1	5.6 (5.2–6.0)		10.0 (9.5–10–6)		17.8 (17.2–18.5)	
Black	4,223	11.9	7.5 (6.7–8.3)		12.0 (11.0–13.0)		20.4 (19.2–21.6)	
Yellow	996	2.8	8.6 (6.9–10.3)		12.9 (10.8–15.1)		23.4 (20.7–26.0)	
Brown	16,841	47.3	7.0 (6.7–7.4)		11.6 (11.1–12.1)		19.4 (18.8–20.0)	
Indigenous	315	0.9	9.7 (6.3–12.9)		15.8 (11.6–19.9)		26.6 (21.6–31.6)	
Marital status				< 0.001		< 0.001		< 0.001
Without partner	8,881	24.8	9.3 (8.7–9.9)		15.0 (14.2–15.7)		22.7 (21.8–23.6)	
With partner	26,963	75.2	5.8 (5.5–6.1)		10.0 (9.6–10.6)		18.0 (17.5–18.5)	
Number of residents				< 0.001*		0.001*		< 0.001*
1–3	15,389	43.0	7.0 (6.6–7.4)		11.5 (11.0–12.0)		20.0 (19.4–20.6)	
4–5	15,147	42.4	5.8 (5.4–6.1)		9.9 (9.4–10.4)		18.0 (17.4–18.6)	
6 or more	5,216	14.6	8.1 (7.4–8.9)		13.8 (12.8–14.7)		19.9 (18.8–21.0)	
*Bolsa Família* Program				0.36		0.06		0.004
No	18,974	52.9	6.5 (6.2–6.9)		10.9 (10.4–11.3)		19.7 (19.1–20.3)	
Yes	16,870	47.1	6.8 (6.4–7.2)		11.5 (11.0–12.0)		18.5 (17.9–19.1)	
Paid work				< 0.001		< 0.001		< 0.001
No	24,104	67.3	7.1 (6.8–7.4)		12.0 (11.6–12.4)		19.9 (19.4–20.4)	
Yes	11,740	32.8	5.7 (5.3–6.1)		9.5 (8.9–10.0)		17.7 (17.0–19.0)	
Income *per capita* (in quartiles)				< 0.001*		< 0.001*		< 0.001*
Up to 104	6,160	20.6	8.7 (8.0–9.4)		14.0 (13.1–14.9)		20.3 (19.3–21.3)	
105 to 188	5,737	19.2	6.4 (5.8–7.1)		10.8 (10.0–11.7)		18.0 (17.0–19.0)	
189 to 300	6,561	22.0	6.0 (5.4–6.5)		10.8 (10.0–11.5)		18.7 (17.7–19.6)	
301 or more	11,395	38.2	5.6 (5.2–6.1)		9.8 (9.2–10.3)		18.8 (18.0–19.5)	
Adequacy of the structure				< 0.001		< 0.001		< 0.001
Inadequate	2,320	6.6	8.9 (7.8–10.1)		13.6 (12.2–15.1)		22.8 (21.1–24.5)	
Less adequate	19,727	56.3	6.8 (6.4–7.2)		11.4 (11.0–11.9)		19.9 (19.3–20.4)	
Adequate	12,975	37.1	5.8 (5.4–6.2)		10.1 (9.6–10.6)		17.0 (16.3–17.6)	
Adequacy of the process				< 0.001		< 0.001		< 0.001
Inadequate	3,867	11.2	8.1 (7.3–9.0)		13.2 (12.1–14.3)		24.0 (22.6–25.3)	
Less adequate	19,211	55.5	6.7 (6.3–7.0)		11.4 (10.9–11.8)		19.8 (19.2–20.4)	
Adequate	11,537	33.3	5.8 (5.4–6.3)		9.9 (9.3–10.4)		15.8 (15.1–16.5)	

PMAQ: Program for Improving Access and Quality of the Primary Care; MHDI: Municipal Human Development Index; FHS: Family Health Strategy

* p-value: Chi-square test for linear trend.

Approximately 50.0% of the interviewees lived in municipalities with MHDI of up to 0.75, 63.2% lived in small and medium-sized municipalities (with up to 100,000 inhabitants), 58.6% lived in municipalities with over 65.0% of FHS coverage, and 73.8% lived in the Northeastern and Southeastern States. Regarding the demographic and socioeconomic characteristics, most were aged between 25 and 35 years (37.4%), were brown (47.3%), lived with partner (75.2%), lived in households with up to three persons (43.0%), did not receive *Bolsa Família* (52.9%), had no paid work (67.3%), and had per capita income greater than R$189,00 (60.2%). In relation to the characteristics of the health unit, 37.1% and 33.3% of the women were interviewed in units with adequate structure and process for examination, respectively (Table 1).

When we individually look at the materials and inputs that made up the variable of adequate structure, we identified that 94.6% of the women were interviewed at a BHU that had gynecological exam table, 95.3% had spotlight for gynecological examination, 94.0% had brush for the collection of Pap smear, 85.2% had disposable speculum, 94.2% had Ayre spatula, 80.0% had slide clamp, 70.3% had plastic bottle with lid, 93.5% had frosted glass slide, 74.4% had gloves, and 92.2% had record to request Pap smear. Regarding the work procedure of the teams of the BHU, 97.4% of the women were present in services that collected Pap smear, 82.6% had the record with the number of women with altered examination, 87.7% carried out the follow-up of women after treatment, 92.4% used dissemination and awareness strategies for Pap smear, 82.6% provided educational and health promotion actions focused on cervical cancer, and 44.2% had the record of the number of women with late collection.

Of the total number of users studied, 28,279 (79.4%) reported having had Pap smear at the BHU where they were interviewed, and 4,970 (14.0%) reported not performing it at that BHU. Of the latter ones, 1,557 (31.3%) had the test at another BHU, 527 (10.6%) reported doing so in a hospital, 1,672 (33.6%) in private practice, and 315 (6.3%) in another location.

In addition, 2,368 women reported never having carried out cervical cancer screening, resulting in a prevalence of lack of access throughout life of 6.7% (95%CI 6.4–6.9). Among those interviewed, the frequency of late examinations (over 36 months) was 11.2% (95%CI 10.8–11.5), and the lack of guidance was reported by 19.2% of the women (95%CI 18.7–19.6) (Figure).

**Figure:**
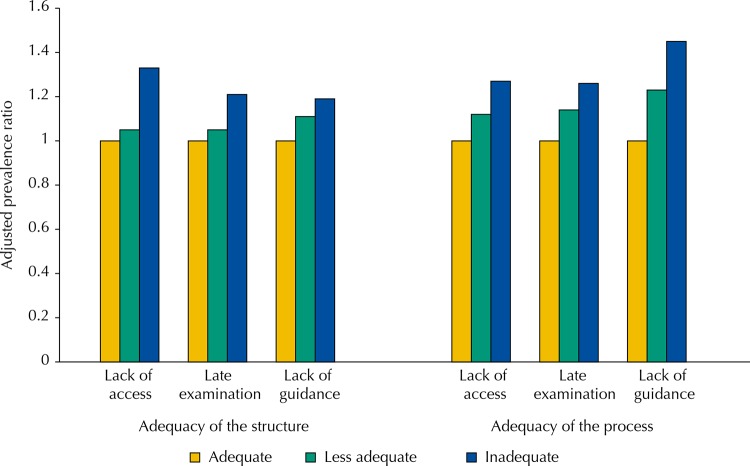
Quality of the adequacy of the work structure and process in the units of the Family Health Strategy. PMAQ, Brazil, 2012.

In the bivariate analysis, the outcomes of lack of access throughout life, last exam being over 36 months ago, and lack of guidance on the examination showed linear increase trend, as the HDI of the municipalities decreased (Table 1). In the adjusted analysis (Table 2), lack of access throughout life, late examination, and the lack of guidance were 2.42, 1.85, and 1.35 times higher, respectively, in poorer municipalities (MHDI up to 0.658) compared to richer municipalities (MHDI above 0.806).

**Table 2:** Crude and adjusted analysis between outcomes and contextual and individual variables and the variables of characteristics of health services. PMAQ, Brazil, 2012.

Variable	Lack of access throughout life		Examination carried out more than 36 months ago		No guidance on the examination	
		
	Crude analysis	Adjusted analysis	Crude analysis	Adjusted analysis	Crude analysis	Adjusted analysis
					
	PR (95%CI)	PR (95%CI)	PR (95%CI)	PR (95%CI)	PR (95%CI)	PR (95%CI)
MHDI	p < 0.001*	p < 0.001*	p < 0.001*	p < 0.001*	p = 0.009*	p < 0.001*
Up to 0.658	1.84 (1.64–2.07)	2.42 (1.89–3.09)	1.43 (1.31–1.56)	1.85 (1.54–2.22)	1.08 (1.02–1.15)	1.35 (1.18–1.54)
0.659 to 0.75	1.49 (1.32–1.68)	1.73 (1.43–2.11)	1.22 (1.11–1.33)	1.40 (1.21–1.62)	1.11 (1.05–1.19)	1.25 (1.12–1.38)
0.751 to 0.806	1.23 (1.08–1.39)	1.39 (1.19–1.62)	1.14 (1.04–1.25)	1.27 (1.13–1.42)	1.09 (1.02–1.16)	1.20 (1.11–1.30)
0.807 to 0.919	1.00	1.00	1.00	1.00	1.00	1.00
Population size (inhabitants)	p < 0.001*	p = 0.002*	p = 0.001*	p < 0.001*	p < 0.001*	p < 0.001*
Up to 30,000	1.00	1.00	1.00	1.00	1.00	1.00
30,001 up to 100,000	0.93 (0.84–1.03)	1.07 (0.96–1.20)	1.00 (0.92–1.08)	1.12 (1.03–1.22)	1.07 (1.01–1.13)	1.14 (1.07–1.22)
100,001 up to 500,000	0.78 (0.69–0.87)	1.14 (0.98–1.33)	0.90 (0.82–0.98)	1.21 (1.08–1.36)	1.12 (1.06–1.19)	1.35 (1.25–1.47)
500,001 or more	0.78 (0.70–0.88)	1.42 (1.17–1.73)	0.88 (0.81–0.97)	1.33 (1.15–1.54)	1.13 (1.06–1.20)	1.49 (1.35–1.65)
FHS coverage (%)	p < 0.001*	p = 0.084	p < 0.001*	p = 0.001*	p = 0.085	p < 0.001*
Up to 29.9	1.00	1.00	1.00	1.00	1.00	1.00
30.0 to 64.9	1.03(0.87–1.22)	1.04 (0.88–1.24)	1.08 (0.95–1.23)	1.10 (0.97–1.26)	0.98 (0.90–1.07)	1.01 (0.92–1.10)
65,0 or more	1.32(1.13–1.55)	1.17 (0.98–1.41)	1.26 (1.11–1.42)	1.24 (1.08–1.43)	1.03 (0.95–1.12)	1.18 (1.07–1.29)
Geographic region	p < 0.001	p < 0.001	p < 0.001	p < 0.001	p < 0.001	p < 0.001
Midwest	1.86 (1.54–2.24)	1.58 (1.30–1.91)	1.49 (1.29–1.71)	1.34 (1.16–1.55)	1.47 (1.33–1.63)	1.37 (1.24–1.52)
North	1.69 (1.39–2.53)	1.21 (0.97–1.51)	1.26 (1.08–1.46)	1.01 (0.85–1.20)	1.39 (1.25–1.54)	1.22 (1.08–1.37)
Northeast	1.66 (1.44–1.90)	0.97 (0.81–1.17)	1.31 (1.19–1.45)	0.90 (0.78–1.03)	1.22 (1.13–1.31)	1.01 (0.91–1.12)
Southeast	1.25 (1.08–1.44)	1.13 (0.97–1.30)	1.14 (1.03–1.26)	1.06 (0.95–1.18)	1.14 (1.06–1.22)	1.05 (0.97–1.13)
South	1.00	1.00	1.00	1.00	1.00	1.00
Age (years)	p < 0.001	p < 0.001	p < 0.001	p < 0.001	p < 0.001	p < 0.001
25–35	1.74 (1.57–1.92)	1.74 (1.55–1.95)	1.33 (1.24–1.44)	1.32 (1.21–1.44)	1.24 (1.18–1.31)	1.25 (1.19–1.32)
35–49	1.00	1.00	1.00	1.00	1.00	1.00
50–64	1.38 (1.24–1.54)	1.22 (1.07–1.39)	1.35 (1.25–1.46)	1.22 (1.12–1.34)	1.22 (1.15–1.29)	1.12 (1.06–1.19)
Race	p < 0.001	p = 0.002	p < 0.001	p = 0.008	p < 0.001	p < 0.001
White	1.00	1.00	1.00	1.00	1.00	1.00
Brown	1.26 (1.15–1.38)	1.11 (1.00–1.24)	1.16 (1.08–1.24)	1.08 (1.00–1.17)	1.09 (1.04–1.14)	1.02 (0.97–1.08)
Black	1.34 (1.18–1.52)	1.24 (1.07–1.44)	1.20 (1.09–1.32)	1.12 (1.00–1.26)	1.14 (1.07–1.23)	1.09 (1.02–1.18)
Yellow	1.54 (1.24–1.91)	1.43 (1.13–1.82)	1.29 (1.08–1.53)	1.30 (1.07–1.57)	1.31 (1.16–1.48)	1.24 (1.10–1.40)
Indigenous	1.72 (1.22–2.44)	1.48 (1.01–2.18)	1.57 (1.20–2.05)	1.44 (1.07–1.93)	1.49 (1.23–1.80)	1.33 (1.09–1.63)
Marital status	p < 0.001	p < 0.001	p < 0.001	p < 0.001	p < 0.001	p < 0.001
Without partner	1.61 (1.49–1.75)	1.63 (1.48–1.80)	1.51 (1.41–1.60)	1.50 (1.39–1.61)	1.26 (1.20–1.32)	1.24 (1.18–1.30)
With partner	1.00	1.00	1.00	1.00	1.00	1.00
Number of residents	p < 0.001	p = 0.015	p < 0.001	p < 0.001	p < 0.001	p = 0.016
1–3	1.00	1.00	1.00	1.00	1.00	1.00
4–5	0.83 (0.76–0.90)	0.87 (0.78–0.96)	0.86 (0.81–0.92)	0.92 (0.84–0.99)	0.90 (0.86–0.94)	0.94 (0.88–0.99)
6 or more	1.16 (1.04–1.29)	1.01 (0.88–1.16)	1.20 (1.10–1.30)	1.13 (1.02–1.26)	0.99 (0.93–1.06)	0.95 (0.88–1.03)
*Bolsa Família* Program	p = 0.361	p < 0.001	p = 0.056	p = 0.187	p = 0.004	p < 0.001
No	0.96 (0.89–1.04)	1.26 (1.13–1.40)	0.94 (0.89–1.00)	1.06 (0.97–1.15)	1.07 (1.02–1.11)	1.13 (1.07–1.19)
Yes	1.00	1.00	1.00	1.00	1.00	1.00
Paid work	p < 0.001	p = 0.001	p < 0.001	p < 0.001	p < 0.001	p < 0.001
No	1.24 (1.14–1.36)	1.19 (1.08–1.31)	1.27 (1.19–1.36)	1.24 (1.14–1.33)	1.12 (1.07–1.18)	1.12 (1.07–1.18)
Yes	1.00	1.00	1.00	1.00	1.00	1.00
Income *per capita* (in quartiles)	p < 0.001*	p < 0.001*	p < 0.001*	p < 0.001*	p = 0.010*	p = 0.460
Up to 104	1.55 (1.39–1.73)	1.31 (1.15–1.50)	1.43 (1.31–1.56)	1.21 (1.10–1.34)	1.09 (1.02–1.16)	1.06 (0.98–1.14)
105–188	1.14 (1.01–1.29)	1.13 (0.98–1.32)	1.11 (1.01–1.22)	1.06 (0.95–1.19)	0.96 (0.90–1.03)	1.01 (0.93–1.09)
189–300	1.06 (0.94–1.20)	1.02 (0.89–1.16)	1.10 (1.01–1.21)	1.06 (0.96–1.17)	1.00 (0.94–1.06)	1.01 (0.95–1.08)
301 or more	1.00	1.00	1.00	1.00	1.00	1.00
Adequacy of the structure	p < 0.001*	p = 0.009*	p < 0.001*	p = 0.018*	p < 0.001*	p < 0.001*
Inadequate	1.55 (1.34–1.80)	1.33 (1.12–1.58)	1.35 (1.20–1.51)	1.21 (1.06–1.39)	1.34 (1.23–1.46)	1.19 (1.09–1.30)
Less adequate	1.18 (1.08–1.29)	1.05 (0.95–1.17)	1.13 (1.06–1.20)	1.05 (0.97–1.13)	1.17 (1.12–1.23)	1.11 (1.05–1.17)
Adequate	1.00	1.00	1.00	1.00	1.00	1.00
Adequacy of the process	p < 0.001*	p = 0.001*	p < 0.001*	p < 0.001*	p < 0.001*	p < 0.001*
Inadequate	1.40 (1.23–1.59)	1.27 (1.10–1.48)	1.34 (1.21–1.48)	1.26 (1.12–1.41)	1.52 (1.41–1.63)	1.45 (1.35–1.56)
Less adequate	1.15 (1.05–1.26)	1.12 (1.01–1.24)	1.15 (1.08–1.24)	1.14 (1.05–1.23)	1.25 (1.19–1.32)	1.23 (1.16–1.29)
Adequate	1.00	1.00	1.00	1.00	1.00	1.00

PMAQ: Program for Improving Access and Quality of the Primary Care; MHDI: Municipal Human Development Index; FHS: Family Health Strategy; PR: prevalence ratio

* p-value: Wald test for linear trend.

With the increase in the population size of the municipality there was also a decrease in the proportion of lack of access throughout life and examination carried out more than 36 months ago, but there was an increase in the lack of guidance on the examination (Table 1). However, in the adjusted analysis, we observed a change in the pattern identified, with a significant increase of the three outcomes with the increase in population size (Table 2).

Regarding the Family Health coverage in the municipality, the lack of access throughout life and examination carried out more than 36 months ago was greater in the municipalities with greater coverage; on the other hand, lack of guidance had no significant difference (Table 1). When performing the adjusted analysis, lack of access throughout life did not keep the association with FHS coverage, late examination kept the pattern of the gross analysis, and lack of guidance on the examination showed an increase of 18% in the stratum of greater coverage (Table 2).

The South and Southeast regions showed improved performance for the quality indicators of cervical cancer screening compared to the Midwest, Northeast, and North regions (Table 1). In the adjusted analysis, the lack of access throughout life and late examination were significantly higher in the Midwest region, which also showed a greater probability of lack of guidance on the examination, together with the North region, when compared to the other regions (Table 2).

The proportions of lack of access throughout life, examination carried out over 36 months ago, and lack of guidance on the examination were significantly lower in users aged 35 to 49 years, when compared to those aged 25 to 35 years and 50 to 64 years (Table 1). This pattern remained unchanged in the adjusted analysis, with the worst quality indicators of cervical cancer screening among younger women (25–35 years) (Table 2).

White users had the lowest prevalence of lack of access throughout life, examination carried out more than 36 months ago, and lack of guidance on the examination, in contrast with self-reported indigenous or yellow users, who presented the worst situation for the three indicators, and brown and black women were in an intermediate position (Table 1). This pattern was kept in the adjusted analysis (Table 2).

Users with no partner, no paid work, and with lower per capita household income presented the worst prevalence for the three outcomes (Table 1). In the adjusted analysis, this pattern was kept in relation to marital status and paid work. In relation to per capita income, the association was no longer significant for lack of guidance on the examination (Table 2). Users living in households with four to five residents had lower prevalence for the three outcomes in both the crude and adjusted analyzes.

In relation to *Bolsa Família*, the lack of access throughout life was not associated in the crude analysis, but it became significantly greater in the adjusted analysis among users who did not receive the benefit. Late examination did not show an association with *Bolsa Família* either in the crude or in the adjusted analysis. On the other hand, lack of guidance on the examination was significantly higher in users who did not receive the benefit, both for the crude and adjusted analyses.

Lower prevalence for the three quality outcomes of cervical cancer screening was also present among users of services with adequate work structure and process for examination (Tables 1 and 2).

## DISCUSSION

In this study, of the women aged between 25 and 64 years in 2012, users of primary care services in Brazil, 93.3% performed Pap smear at least once in their lives and 88.8% reported an updated examination, reaffirming a significant improvement in the access to cervical cancer screening, already indicated by a previous study^12^. Although the performance of primary care exceeds the parameters of the WHO^26^, the high prevalence of quality indicators for screening does not indicate the full solution of the problems in the country. In addition, our study encompasses users of the FHS, whose performance is better than that of traditional basic services^4^.

The lack of access throughout life, late examination, and lack of guidance vary according to contextual and individual characteristics, in addition to the characteristics of health services, although relatively by little. On the other hand, one in fifteen women aged between 25 and 64 years linked to services of primary health care in Brazil had never performed Pap smear. This missed care opportunity arises from, among other aspects, problems in the work structure and process of the BHU, as previously observed in studies on the performance of FHS^4^ ^,^ ^24^.

The problems in the quality of the screening increased because of the decrease in HDI, a contextual characteristic that expressed the greatest difference in relation to the three outcomes studied. The finding coincides with other Brazilian studies suggesting a lower capacity for health investment in poorer municipalities, which concentrate a larger proportion of vulnerable social groups^6^ ^,^ ^24^. The VIGITEL (2011)^19^ has also found strong positive correlation between the rate of Pap smear at some time in life and in the last three years and the HDI of the municipalities studied.

The increase in population size of the municipality and FHS coverage was associated with an increase of the problems in the quality of the screening. Large urban centers have better economic conditions, greater capacity of investment, and higher qualification of health teams^25^. However, after adjusted analysis, this was not reflected in the quality of cervical cancer screening in the primary health care network, in contrast to findings from population-based studies, such as the World Health Survey (WHS), carried out in Brazil, in 2003. That study included users of the SUS and the private sector, showing a marked social gradient in the coverage of the Pap smear and a lower percentage of examination carried out more than 36 months before, as the population size of the municipalities decreased^22^ ^,^ ^23^. In the crude analysis, our finding was similar to that of the WHS; however, when controlling for the socioeconomic differences between the sizes of municipalities, there was a reversal of the pattern, indicating greater difficulty of large municipalities in ensuring the provision of quality services. Other studies^11^ ^,^ ^25^ also highlight barriers in the performance of primary care in large municipalities resulting from, for example, the urbanization profile. Large social inequalities and poor population concentration can be observed, particularly in metropolitan regions, along with an inequitable distribution and organizational problems of the services, possibly implying longer and costly trips for users.

The increase in the prevalence of poor quality indicators for cervical cancer screening from the increase in municipal FHS coverage is consistent with the findings related to HDI, given the greater presence of this care model in poorer areas and municipalities. A study carried out in 2006 already indicated greater FHS coverage in poorer municipalities, from their orientation for the care of the most vulnerable populations and territories^4^. However, similar to that observed in the adjusted analysis, in which the lack of access throughout life is no longer associated with FHS coverage, investments in the qualification of this model of care show a great potential to promote health equity in Brazil^4^.

The South region had less problems of screening in the primary care network, while the Midwest had more problems, signaling a greater complexity of regional inequalities in the quality of the Pap smear^3^. The finding regarding the better screening quality in the South and Southeast regions when compared to the other areas is coherent with several studies^4^ ^,^ ^5^ ^,^ ^24^. The apparent surprise comes from the worst screening situation in the Midwest, even when compared to the North and Northeast regions. The explanation for the finding is challenging and this study does not exhaust it, but a consideration seems relevant and closely related to the arguments concerning large urban centers. The Midwest is the second most urbanized region in the country, with almost 90% of the population living in cities, and the Federal District (97%) is the Federation Unit with the highest rate of urbanization in the country, together with Rio de Janeiro^8^. The process of urbanization in the Midwest produced a rapid concentration of slums and poor and disorganized areas on the outskirts of the cities of the region, which did not have time to accumulate the infrastructure observed in the municipalities of the South and Southeast regions, including in relation to health equipment^8^ ^,^ ^25^. In addition, the evidence indicates a more equitable pattern in performing Pap smear in the North and Northeast regions, when compared to the Midwest^20^ ^,^ ^21^.

The quality issues of the screening were higher in users aged 25 to 35 years and 50 to 64 years. Martins et al.^14^ highlight that younger women do not perform the examination as frequently as older women. Thus, focus on this group would produce a historic effect of increased coverage of the screening. The incidence of cervical cancer increases between 30 and 39 years of age, reaching its peak from the fifth decade of life, which should guide the initiatives of the BHU to perform screening, especially among older women. In Brazil, Amorim et al.^1^ have analyzed the prevalence of no Pap smear testing according to socioeconomic and demographic variables and the variables related to health behaviors, in women aged 40 years or more, living in the municipality of Campinas, State of São Paulo, Brazil, highlighting the age group of 40 to 59 years as one of the factors associated with no examination.

White women had the lowest prevalence for the three indicators of low quality of cervical cancer screening, even after the adjusted analysis, coinciding with several studies carried out in Brazil^1^ ^,^ ^10^ ^,^ ^14^. The reasons underlying the relative advantage of white users in relation to the other races may be related to adjustment, given the difficulty of accurately measuring family income. In addition, they may be related to cultural and behavioral characteristics related to both users (adherence to screening) and services (different reception of users based on race). Women with a partner who lived in households with four or five persons also had lower prevalence for the three outcomes, similar to that observed in other studies^1^ ^,^ ^10^. These variables are also markers of socioeconomic conditions and the conditions of facilities or support for the examination.

Women who received the *Bolsa Família* benefit had lower prevalence of lack of access for the examination and lack of guidance. The benefit seems to positively influence the access to and quality of the examination, similar to the positive effect found in the reduction of infant mortality^18^. This result indicates a possible compensatory effect of this income transfer strategy in favor of equity, since the women who receive the benefit present greater social vulnerability^13^.

In this study, users with paid work and living in households with higher per capita income had fewer problems in the quality of the screening, indicating social inequity in cervical cancer prevention in primary health care. A study conducted based on data from the *Pesquisa Nacional por Amostra de Domicílios* (PNAD – National Household Sample Survey) of 2008 confirmed the higher number of Pap smear examinations in higher income quintiles^21^. Socioeconomic inequality in the access to the examination was also observed in other publications^11^ ^,^ ^16^.

In addition, the quality of the screening by Pap smear improved according to the adequacy of the work structure and process in the BHU. The results of the assessment of the quality of cervical cancer screening in Brazil reaffirm the relevance of adequate work structures and processes to improve the performance of services, as classically highlighted by Donabedian^2^. In this sense, investments in the primary care network are essential not only to improve the performance of services, but also to promote social equity in health, considering that the adequacy of the work structure and process reduced the probability of occurrence of all indicators of low quality for cervical cancer screening. This is certainly one of the most relevant findings of the study, considering its potential to subsidize policies aimed at improving the prevention of cervical cancer and its meaning for more equitable health conditions for the population that uses basic health services, particularly the FHS. The finding is also relevant to guide the training and qualification of health professionals in undergraduate and graduate courses.

Among the limitations of this study, we highlight the assessment of health teams that voluntarily joined the PMAQ, which may overestimate the performance of services compared to other health teams. The location of the users interviewed in the health team itself suggests that the prevalence of low quality could be higher in the case of a population-based assessment. On the other hand, the voluntary adherence of teams allowed us to know the possible standard of excellence of the Brazilian primary health care, being a parameter for future comparisons in the next PMAQ cycles, in relation to other services of the SUS or the private sector. We also highlight the great magnitude of the sample of users and the national scope of the study, allowing comparisons between the regions, municipalities, and characteristics of users. The percentage of information loss for the studied variables was small, except for the variable of per capita income, thus reinforcing the internal validity and facilitating a more detailed comparison of the three selected outcomes for the characterization of the problems in the quality of cervical cancer screening.

The quality of the Pap smear, although improved with the adequacy of the work structure and process in the BHU, strongly depends on the social characteristics of the context and the population studied. It is worth highlighting the greater access and greater guidance for the examination in beneficiaries of the *Bolsa Família* Program. In short, investments in both the work structure and process of the BHU, as well as in social programs of income transfer and progress of social conditions of the population, are essential to improve the quality of the program of cervical cancer screening in Brazil.

## Footnotes

a Ministério da Saúde (BR), Secretaria de Ciência, Tecnologia e Insumos Estratégicos, Departamento de Ciência e Tecnologia. Agenda nacional de prioridades de pesquisa em saúde. 2. ed. Brasília (DF): Ministério da Saúde; 2011 [cited 2016 Feb 27]. (Série B. Textos Básicos em Saúde). Available from: http://bvsms.saude.gov.br/bvs/publicacoes/agenda_nacional_prioridades_2ed_3imp.pdf

b Ministério da Saúde (BR), Secretaria de Atenção à Saúde, Departamento de Atenção Básica. Programa Nacional de Melhoria do Acesso e da Qualidade da Atenção Básica (PMAQ). Instrumento de avaliação externa do Saúde Mais Perto de Você – acesso e qualidade. Brasília (DF): Ministério da Saúde; 2012 [cited 2016 Feb 27]. (Série A. Normas e Manuais Técnicos). Available from: http://bvsms.saude.gov.br/bvs/publicacoes/instrumento_avaliacao_saude_mais_perto_voce_acesso_qualidade.pdf

c Ministério da Saúde (BR), Secretaria de Atenção à Saúde, Departamento de Atenção Básica. Controle dos cânceres do colo do útero e da mama. 2.ed. Brasília (DF): Ministério da Saúde; 2013 [cited 2016 Feb 27]. (Cadernos de Atenção Básica, 13). Available from: http://dab.saude.gov.br/portaldab/biblioteca.php?conteudo=publicacoes/cab13
